# Challenges and future of precision medicine strategies for breast cancer based on a database on drug reactions

**DOI:** 10.1042/BSR20190230

**Published:** 2019-09-06

**Authors:** Xiping Zhang, Hongjian Yang, Ruiping Zhang

**Affiliations:** 1 Department of Breast Surgery, Cancer Hospital of University of Chinese Academy of Sciences, Zhejiang Cancer Hospital, Hangzhou 310022, Zhejiang Province, China; 2 Shanxi Academy of Medical Sciences, Radiology Department of Shanxi Dayi Hospital, Taiyuan, 030032, Shanxi Province, China

**Keywords:** breast cancer, database, drug reaction, precision medicine, target, transcriptome

## Abstract

Breast cancer (BC) is a malignancy with the highest incidence in women. Great progress has been made in research related to traditional precision medicine for BC. However, many reports have suggested that patients with BC have not benefited a lot from such progress. Thus, we analyze traditional precision medicine strategies for BC, sum up their limitations and challenges, and preliminarily propose future orientations of precision medicine strategies based on a database on drug reaction of patients with BC. According to related research, traditional precision medicine strategies for BC, which are based on molecular subtypes, perform pertinent treatments, new drug research and development according to molecular typing results. Nevertheless, these strategies still have some deficiencies. First, there are very few patients with each molecular subtype, the match ratio of drugs is low. Second, these strategies can not solve the problem of poor drug sensitivity resulting from heterogeneity. The main strategy we put forward in the present paper is based on patients’ varying drug reactions. Focusing on treating existing patients and maximizing the utilization of existing drugs, it is expected to not have deficiencies of traditional precision medicine for BC, including low match rate and poor therapeutic efficacy arising from tumor heterogeneity of BC.

## Introduction

In 2015, Obama, who served as President of the United States, first launched the precision medicine initiative in his annual message. He declared that 0.215 billion U.S. Dollars would be raised for research and practices of precision medicine initiatives [1]. Subsequently, China promulgated corresponding precision medicine strategies, planning to invest RMB 60 billion in tumor diagnosis and treatment in succession by 2030. Besides, precision medicine is proposed to be listed as one of major programs in research on development of health and healthcare in the ‘13th five-year plan’ [2]. At present, breast cancer (BC) has become a malignancy with the highest incidence in Chinese women, and some experts predict that there would be 2500000 patients with BC in 2021 [3]. Hence, performing research on precision medicine for BC is quite favorable for improving treatment outcomes of BC, reducing mortality and increasing survival rate. Recently, there have been some reports about BC and precision medicine [4,5], which have mainly reviewed precision medicine from the perspective of traditional molecular subtypes and target mutated genes for the purpose of providing references for clinical practices in the treatment of BC. Lately, beneficiaries of precision medicine strategies with traditional molecular subtypes have been greatly questioned. Some scholars consider that the roles of precision medicine technologies in guiding clinical practices are rather uncertain.

Traditional precision medicine strategies for BC, which are based on molecular subtypes, perform pertinent treatments, new drug research and development according to molecular typing results. Nevertheless, these strategies still have some deficiencies. First, there are very few patients with each molecular subtype, the match ratio of drugs is low. Except for triple-negative BC (TNBC), the other molecular subtypes of BC may have different drug sensitivities because of different receptor expression levels. For example, the expression levels of estrogen receptor (ER) and PR receptors in Luminal A will be the most changeful. For example, ER is positive from 1 to 100%, but the sensitivity of 1 and 100% ER positive patients to endocrine therapy is certainly different. In the process of neoadjuvant endocrine therapy, this confusion will be faced more. Second, these strategies can not solve the problem of poor drug sensitivity resulting from heterogeneity. In the clinic, we often encounter patients with drug resistance to endocrine therapy, chemotherapy or targeted therapy. Once drug resistance occurs, it will significantly affect the efficacy of patients. Drug resistance has also prompted us to re-recognize individual treatment strategies for BC patients with different molecular subtypes from the perspective of precision medicine, and to further explore them from a micro perspective. The main strategy we put forward in the present paper is based on patients’ varying drug reactions. Focusing on treating existing patients and maximizing the utilization of existing drugs, it is expected to not have deficiencies of traditional precision medicine for BC, including low match rate and poor therapeutic efficacy arising from tumor heterogeneity of BC.

Therefore, the present paper analyzes the limitations and challenges of traditional precision medicine strategies based on molecular subtypes. Taking BC for example, it illustrates future orientations of individualized therapies from a new perspective.

## Limitations of precision medicine strategies for BC based on molecular subtypes

In 2013, BC was classified into luminal A BC (ER+, PR ≥ 20%, Her-2-, Ki-67 < 20%), luminal B BC (ER+, PR < 20% and/or Her-2+ and/or Ki-67 ≥ 20%, Her-2-overexpression (ER-, PR-, Her-2+), and basal-like (ER-, PR-, Her-2-) BC [6]. After the era of precision medicine, patients with BC will be subdivided into subtypes more precisely. Concerning precision medicine, the main strategy is to first find pathogenic genes of different tumors, based on which patients’ molecular subtypes are further divided. At last, the precise targeting therapies are performed on the basis of subtyping results. In view of deficiencies in treatment, new drugs are researched and developed.

Over the years, much progress has been made in looking for pathogenic genes in precision medicine for BC. Nik-Zainal et al. [7] reported in the *Nature* that they analyzed information of all genomes in 560 patients with BC and discovered 93 potential tumor-related gene mutations. The authors thought that the research outcomes covered all related genes which would cause BC. Once any of these 93 genes mutated, BC would be caused [7]. In addition, Ko et al. discovered a new pathogenic gene known as Genomic Transcript 198 (GT198) in 254 patients with BC before and after menopause [8]. Under normal circumstances, GT198 is regulated by estrogen. It is also a co-activator of ERs. After this gene mutates, GT198 will be able to promote tumor growth without estrogen. According to Kwong et al.’s [9] research on Chinese women, RECQL (RecQ protein-like) gene mutations are directly associated with higher risks of BC, as a result of which the new genetic BC mutation was detected. In a large-scale global study, Southey et al. [10] detected the mutation of PALB2 (partner and localizer of BC susceptibility gene 2), CHEK2 (checkpoint kinase 2), and ATM (ataxia telangiectasia-mutated gene), their study was deemed to be the world’s first research which demonstrated that rare genetic mutations also contributed to relatively high risks of BC. It is beyond doubt that these studies have laid a solid foundation for further molecular subtype of precision medicine for BC. However, these research strategies have problems such as significant investments, long period, relatively low marginal revenues and ineffective treatment resulting from tumor heterogeneity. Marginal revenues refer to revenues from the increase in a product. Since the cost for developing a new drug is extremely high and only a minority of patients are under certain targeted molecular subtype of the drug, the marginal revenues have declined.

According to a review published in the magazine named *Nature* in September 2016, only a small number of people benefit from precision medicine, which is meaningless for most tumor patients [11]. It was mentioned that MD Anderson Cancer Center found in gene sequencing of 2600 people that only 6.4% people could benefit from targeted drugs [12]. According to the data about matching plans of the National Cancer Institute (NCI), only 2% people can benefit from targeted drugs. Prasad [11], who is a reviewer, considers that it is inspiring to customize therapies with precision strategy. However, at present, it is neither feasible nor cost-effective to select targeted drugs and perform treatment with these drugs by acquiring patients’ genetic information. In addition, it is impossible to guarantee the success of these practices in the future.

Thereafter, Hunter [13] pointed out in the *New England Journal of Medicine* that the benefits from treating patients only by molecular techniques were still unknown. BC was specially mentioned in that paper, where they thought that traditional clinical predictors were generally indicators based on tumor size, stage, and lymph node status. The changes in expressions of BC genes which must be detected for precision medicine, to a greater extent, predict whether tumors are recurrent. Nonetheless, no direct evidence has proven whether such changes are associated with therapeutic effects or not. It was even pointed out that targeted drugs used in precision medicine could not change tumorigenesis, but could only improve patients’ quality of life and give people an illusion that these drugs were useful for treating tumors; these disputes posed challenges to the development of precision medicine [13]. Tannock and Hickman [14] also expressed their views in the same journal. First of all, they confirmed that development of sequencing technologies promoted research on molecular characteristics of tumors. Benefiting from this confirmation, trastuzumab, which is a targeted drug for Her-2 of BC, has turned into a conventional drug. However, individualized medicine brings limited benefits as revealed from current outcomes of clinical trials and research; tumor cells would become tolerant to single targeted drugs and thus activate other pathways; at present, targeted drugs only target some gene products of signaling pathways [14]. Take BC for example, clinical research data suggested that after analyzing 423 women with BC by comparing genomics and gene sequencing, current drugs for precision medicine were found to be only suitable for 13% patients. However, only 3% patients had drug reactions at last, which was close to data of American NCI’s match initiative mentioned above [15].

## Challenges to precision medicine for BC

To better put forward countermeasures and future development orientations, we first analyzed these problems. Currently, precision medicine mainly encounters two difficulties. On one hand, the match ratio is very low because there are too many mutations of pathogenic genes causing BC. There are more than 100 pathogenic genes of BC at least, and the mutation of these genes would contribute to the development of BC. For precision medicine, molecular subtypes of patients with BC must be divided. As a consequence, it is inevitable that there are only a small number of patients with a molecular subtype. Under this circumstance, tumor drugs selected are only applicable to this small proportion of people. Thus, costs are enormous and yields are extremely low. On the other hand, tumors are heterogeneous: tumors are not single diseases. The mutations of cancer cells are partially the same in a tumor because they are related to clone. However, these cells are continuously exposed to different pressures such as tumor microenvironment and iatrogenic measures during tumor development. In this case, cancer cells constantly have new mutations to adapt to pressure so that the posterity can obtain survival advantages. Owing to such selective pressure and mutation, abnormal phenomena of cancer cell genomes, including primitive and new mutations, have gradually appeared in the course of tumor progression. As a consequence, subclonal diversity and heterogeneity exists apart from the main clones inside tumors. Heterogeneity of BC exists in different time and space such as primary foci, therapeutic processes, and metastatic lesions. Just like aforementioned views of Tannock and Hickman [14], certain specific drug for precision medicine can only kill a kind of mutant clonal cell lines, whereas other new mutant clonal cell lines which can not be killed begin to amplify; the mutant cell lines which were targeted would activate other survival pathways and thus making drugs for precision medicine inactive or generate drug resistance. Furthermore, Huang et al. [16] discovered in their research that lapatinib was effective for treating patients with Her-2-positive BC. However, long-term medication leads to activation of molecular pathways such as interleukin-6 (IL-6), signal transducer and activator of transcription 3 (STAT3), thereby generating tolerance to lapatinib [16]. In other words, drugs for precision medicine can not kill other new clonal cell lines after killing host clonal cells, perhaps because they can not block the creation of tumor micro-environment. Hence, we confirm that mutation is just a cause of tumors, but not the sole one.

## Orientations of precision medicine for BC

In view that BC has something in common with other cancers, the similar therapeutic strategies of precision medicine are not only applicable to BC, but also are expected to be applied in the field for all tumors.

First, the important thing is how the BC patients shall be classified. In our opinion, judging BC patients’ therapeutic reactions to different drugs with big data are likely to become a future research direction. We discovered that medicine is generally divided into symptom-based empirical medicine, evidence-based medicine based on the limited pathological/physiological indicators, and precision medicine based on multiple omic molecular indicators. The ultimate purpose of precision medicine is to maximize therapeutic efficacy. We always simultaneously consider data about BC patients’ physiological features, pathological features, and drug reactions so that eligible patients can be treated by corresponding drugs and therapeutic efficacy can be improved accordingly. It is necessary to recommend which drugs shall be used for BC patients. Precision medicine is not only based on individuals’ experience, but also based on the patients’ pathological and physiological indicators which are connected with drug reactions from the multiple centers and big-sample data, in order to maximize therapeutic efficacy.

In other words, connections between patients’ pathological or physiological features and drug reactions are very essential for precision medicine. Although it is undeniable that precision medicine is a future development trend, the existing strategies for precision medicine are questioned owing to their fast development. Then, can we detect pathological and physiological indicators of evidence-based medicine with omics technologies? For instance, omic characteristics or molecular types related to BC patients and drug reactions are screened in place of detecting physiological and pathological indicators such as blood examinations and urine tests. In this way, we can recommend the most suitable drugs to BC patients dependent upon these omic characteristics or molecular types.

In other words, drugs can be recommended based on omic characteristics or molecular types related to drug reactions and post-medication drug reactions rather than pathological/physiological characteristics and post-medication drug reactions which are considered in recommending drugs in traditional clinical practices. If patients’ omic characteristics or molecular types were found before medication that were identical with those corresponding drug reactions, it suggested that their pathological and physiological characteristics had large similar probability. In other words, patients might have similar drug reactions. Thus, we shall recommend the same drugs to these patients. However, it is inadvisable to not recommend these patients to take the same drugs just because of their different molecular subtypes with distinct gene mutations.

We will have a huge case matching database if numerous patients’ omic characteristics or molecular types related to drug reactions and the reactions to treatment by precision medicine are included in a designated database regarding drug reactions by obtaining high-quality research information and comprehensive large data analysis from clinical study. For instance, when a new BC patient requires individualized treatment plan, we can match related data in the database about drug reactions according to this patient’s omic characteristics or molecular types. After successful matching, a precise treatment plan with the best therapeutic efficacy can be recommended to this new patient.

### The restrictive factors of precision medicine

#### Evidence-based medicine and precision medicine

In the age of evidence-based medicine, with the advent of the concept that BC is a systemic disease and the validation of a large number of clinical experimental data, BC patients with different immunohistochemical types have achieved fruitful results in choosing different treatment modalities, such as surgery, chemotherapy, radiotherapy, endocrine therapy, or targeted therapy. It laid the foundation for classification and treatment of BC. However, this treatment model based on evidence-based medicine is not perfect enough, the kind of treatment methods are limited especially for BC patients with high risk of recurrence or drug-resistance. With the concept of precision medicine put forward, these problems can be better solved.

In the era of evidence-based medicine, despite the fruitful achievements in the field of standardized treatment of BC, the imperfect individualized information limits its further development. Precision medicine enriches the connotation of evidence-based medicine because of its more individualized and refined disease management. By obtaining high-quality research information and comprehensive large data analysis, more reliable predictors and treatment targets of BC will be obtained, and then achieving accurate treatment for BC patients.

The concept of precision medicine was first proposed by the U.S.A. National Academy of Sciences in 2011 in order to establish new knowledge networks, promote biomedical and clinical research, and formulate individualized treatment program by assessing patient omics information. Professor Shao, a well-known professor in China, pointed out that the development direction of BC diagnosis and treatment under the background of precision medicine should be include: (1) drawing the omics molecular map; (2) clinical transformation of ctDNA; (3) detection and management of tumor heterogeneity; (4) exploration of the interaction between tumor cells and tumor microenvironment [17].

#### Puzzles in precise medicine of BC

However, the ideal and reality are difficult to match. The implementation of precision medical treatment is restricted by many factors, especially for TNBC. Because of the heterogeneity of BC, even if it is a good design, it is often not ideal in practical application. For example, ER, PR, and Her-2 are all positive patients who were used for endocrine and target therapies, and there will be a mutual interference effect. De Laurentiis et al. [18] have conducted a meta-analysis on the interaction between the response to endocrine treatment and the overexpression of Her-2 in metastatic BC. They found that Her-2-positive metastatic BC is less responsive to any type of endocrine treatment [18].

Despite the use of ER, PR, Ki-67, and Her-2 as biomarkers in treatment, these genes are not among the most frequently mutated in BC. Two of the most prominent genes in BC pathology—the homologous DNA-repair genes *BRCA1* and *BRCA2*—are best known for harboring germline variants associated with hereditary breast and ovarian cancer syndrome [19]. Olaparib is an oral poly(adenosine diphosphate-ribose) polymerase inhibitor that has promising antitumor activity in patients with metastatic BC and a germline BRCA mutation. Among patients with Her-2-negative metastatic BC and a germline BRCA mutation, olaparib monotherapy provided a significant benefit over standard therapy; median progression-free survival was 2.8 months longer and the risk of disease progression or death was 42% lower with olaparib monotherapy than with standard therapy [20]. Olaparib is still in the experimental stage in China and has not been approved for clinical application.

In recent years, the problem of resistance to endocrine therapy in precision medicine has been a hotspot of research. One of the mechanisms that cause resistance of endocrine therapy are mutations on the ER-α (*ESR1*) gene that render the ER from constitutive activation. From the perspective of cancer genomics, luminal A subtypes are shown to have the most mutated genes, with the most frequent in PIK3CA, followed by MAP3K1, GATA3, TP53, CDH1 and MAP2K4 [21]. Everolimus, an mTOR inhibitor, has been shown to increase the efficacy of endocrine therapy and overcome resistance to endocrine therapies. Clinical studies have suggested that everolimus combined with endocrine therapy prolongs progression-free survival in hormone receptor-positive BC patients. However, because BC includes a group of highly heterogeneous tumors, patients may have different responses to everolimus. Numerous preclinical studies have shown that PIK3CA/PTEN mutations are predictive of sensitivity to everolimus [22]. The PI3K/Akt signaling axis contributes to the dysregulation of many dominant features in BC including cell proliferation, survival, metabolism, motility, and genomic instability [23]. Activating mutations of PIK3CA are the most frequent genomic alterations in ER-positive breast tumors, and selective phosphatidylinositol 3-kinase α (PI3Kα) inhibitors are in the clinical development. Searching for mechanisms of resistance, we observed that suppression of PI3K signaling results in induction of ER-dependent transcriptional activity [24]. Targeted therapies specific to PIK3CA-mutated cancer are seeing preliminary success in the preclinical and clinical trials. To target the PI3K pathway is less specifically, such that patients harboring variants in other pathway components, including PIK3R1, AKT genes, or PTEN, might benefit from treatment [25].

Through reading the related literatures, we discovered there is a shortage in the separate precision treatment. When considered the combination or synthesis, we found the results showed an ideal effect in endocrine therapy. Therapeutic strategies against hormonal receptor-positive (HR+)/Her-2 + BC with poor response to trastuzumab need to be optimized. Hsu et al. established two HR+/Her-2+ patient-derived xenograft (PDX) models to explore targeted therapies for Her-2 + BC [26]. Compared with the standard trastuzumab treatment, the present study demonstrates alternative therapeutic strategies against HR+/Her-2 + tumors through establishment of two PDXs coupled with integrative omics analyses and *in vivo* drug efficacy examination [26]. Palbociclib, a CDK4–6 inhibitor, combined with endocrine therapy is a new standard of treatment for HR+ metastatic BC. Du Rusquec et al. [27] present the first real-life efficacy and tolerance data of palbociclib plus fulvestrant in this population. Some patients receiving palbociclib+fulvestrant were prospectively analyzed. In their pretreated population including everolimus, fulvestrant plus palbociclib provides an mPFS of 5.8 months with the same magnitude of benefit for fulvestrant-pretreated patients [27].

### The relationship among of precision treatment for BC, miRNAs and long non-coding RNA, or other related genes

As a small non-coding and single-chain RNA, miRNA has been extensively reported to be involved in regulating multiple signaling pathways in BC. Zhang et al. [28] explored some miRNA biomarkers in the blood of BC patients based on miRNA profiling. Their data supported that the potential of the five identified miRNAs (miR-30b-5p, miR-96-5p, miR-182-5p, miR-374b-5p, and miR-942-5p) can be used as novel biomarkers for the detection of BC, and indicated that they might be involved in BC development and progression. The above-mentioned five miRNAs were significantly enriched in numerous cancer-related pathways, including ‘MicroRNAs in cancer’, ‘Pathways in cancer’, ‘FoxO signaling pathway’, ‘Ras signaling pathway’, ‘Rap1 signaling pathway’, ‘MAPK signaling pathway’, and ‘PI3K-Akt signaling pathway’ [28]. Ectopic expression of miR-205 significantly inhibits cell proliferation and anchorage-independent growth as well as cell invasion. miR-205 may serve as a unique therapeutic target for BC [29]. At present, we have not developed a drug targeted to a certain miRNA for BC, but our research group is already in progress.

Some progress has been made in research of long non-coding RNA (hereunder referred to as LncRNA) related to BC. Lots of data about LncRNA transcription concerning BC have been obtained from large-scale omics research (e.g. transcriptomes and chips). Metastasis-associated in lung adenocarcinoma transcript 1 (MALAT1), an lncRNA that was first recognized as a prognostic parameter for patient survival of stage I lung cancer, is up-regulated in multiple human malignancies, including BC. MALAT1 overexpression was also associated with poor RFS in tamoxifen treated ER-positive BC patients, which might serve as a potential biomarker to predict endocrine treatment sensitivity [30]. The results of Xu et al.’s [31] findings indicated that MALAT1 is a novel regulator of EMT in BC and may be a potential therapeutic target for BC metastasis. LncRNA H19 has been well studied playing an important role in BC progress and the expression of H19 may service as a diagnostic target for BC. Plasma H19 may serve as a potential biomarker for BC early screening and prognosis monitor [32]. Up to now, we have not developed a drug targeted to a certain lncRNA for BC, because of the need for the development of precision medicine, our research group are in the process of doing this arduous task.

Precision medicine is medicine optimized to the genotypic and phenotypic characteristics of an individual. Studying precision medicine involves a systems biology approach that integrates mathematical modeling and biology genomics, transcriptomics, proteomics, and metabolomics [33]. The treatment of TNBC emphasizes enhancing healthcare and developing personalized medicine. Because three receptors of TNBC are all negative and lack of effective therapeutic targets, which has been a difficult point in the field of precision medicine. To respond to this difficult point, the researchers have turned their attention to a different approach to scientific enquiry: the era of ‘big biology’ and the integrative study of biological systems, also called ‘Omics’ technologies. The term omics comprises different fields of molecular studies and characterizes a global view on biological molecules such as DNA, RNA, proteins, and metabolites. Combined ‘omics’ approach offers a major tool for the understanding of a challenging cancer model, TNBC [34].

Which kind of omic technologies (genomics, epigenetics, transcriptomics, and proteomics) shall be used for designing a database for matching drug reaction? The omics technologies can help us gain a better understanding of carcinogenesis [35,36]. Modern ‘omic’ strategies can potentially make a major contribution to meeting this need. Technological advances in the field of nucleic acid sequencing, mass spectrometry, and metabolic profiling have driven progress in genomics, transcriptomics (functional genomics), proteomics, and metabolomics [37].

Precision medicine, which is inseparable from omic detection technologies, is associated with genomes, transcriptomes, proteomes, and latest metabolomic technologies. For genomics, we have discovered that correlations between gene changes and diseases have been questioned; recently, it was published in the *Cell* that multi-gene and even whole-genome sequencing are not quite meaningful for understanding diseases [38]. In addition, proteomics and metabolomics are not so accurate and reliable that literature about precision medicine is still rare.

Transcriptomes are a complete range of products from RNA transcription in certain stage of development or under physiological conditions. The gene transcription in cells is dependent upon type, cycle and environment of cells, so transcriptomes are quite diverse [39,40]. Transcriptomics, aiming at studying transcriptomes, is a discipline for evaluating parallel expression level of genomes. In general, it is acknowledged that modes of gene expression can reflect physiological conditions of cells [41,42]. The advantage of transcriptomics consists of its direct association with responses to environment, including external environment and micro-environment. With high responsiveness, transcriptomics is the most suitable for investigating patients’ drug reactions. Nevertheless, gene mutations are changes accumulated through several years and even decades. Thus, we propose designing a transcriptomics-based database matching drug reaction. In this database, two kinds of information shall be recorded for each case, including patients’ transcriptomic information regarding drug reactions before and after their medication (e.g. recurrence and patients’ survival). If the transcriptomic information related to drug reactions is similar between new patients before medication and old patients recorded in the database, we will conclude that those new patients would have similar drug reactions to the old patients’ and thus recommend the new patients to take similar drugs as those used by the old ones.

Genomics studies investigate differences in sequences in nucleotides that constitute protein coding genes, non-coding DNA, and regulatory regions while proteomics studies identify function of proteins in cancer cells compared with cancer cells. Metabolomics is the newest layer of ‘omics’ data that is rapidly gaining attention of BC researchers worldwide. The metabolome of a cell comprises the highly complex biochemical pathways with numerous small molecules or metabolic substrates that include amino acids, sugars, lipids, and other bioactive agents [37].

## Conclusions and prospects

According to the statistics announced by the China Anti-cancer Association, China is one of countries where the morbidity of BC has increased at the fastest pace. In recent years, the morbidity of BC has progressively increased by 3% year after year. The latest data report on cancer of Chinese cities announced in 2017 suggests that the increase in the mortality of BC is the fastest among cancers, and the age of onset tends to be younger (in China, statistics about cancer are generally presented 3 years later after their generation, so the latest data on incidence and death were announced in 2013) [43]. Regretfully, the quantity of drugs which have been approved and are under investigation as mentioned in above literature is still far from satisfying the demands hidden behind molecular subtypes of BC. We are supposed to take advantage of current drugs available, making great efforts to improve quality of life and survival of patients with BC. We are expected to find out the most suitable treatment plans for present patients based on our clinical experience and drug options.

Apparently, it is extremely uneconomical to screen targets of all patients since several kinds of targeted therapeutic drugs have been available. Even if all targets can be screened, difficulties such as low rate of return and impossibility for prolonging patients’ survival would be still encountered at the time of their medication. In addition, studies on genomes, proteomes, and metabolomics are still incomplete, so we think that attention shall be paid to study how to create a database on patients’ drug reactions based on transcriptomic differences to guide new patients’ choices of drugs. We will be able to have a huge database when patients’ information is accumulated to certain extent. This database will be a cornerstone for developing pertinent plans for treating subsequent new patients through precision medicine.

At last, this strategy will be quite helpful for us to maximize the application of current drugs. In view that our matching is based on omic characteristics or molecular types of patients instead of specific disease classification or certain gene mutation, we are expected to judge that the same drugs have equivalent effects for two patients whose BC tissues have identical pathological and physiological features from the perspective of omics.

Furthermore, it usually shall take decades to develop a kind of new drug. It is beyond doubt that this strategy can bring us considerable benefits, because it makes it possible for us to ‘use old drugs for new purposes’. For instance, Cook et al. [44] have discovered that chloroquine, as antimalarial drug, delays and even reverses resistance to antiestrogens like tamoxifen when it is used by patients with BC whose ERs are positive. Chauhan et al. [45] have found that losartan is helpful for supporting the treatment of BC as hypertensive drug. Recently, it was reported in *Proceedings of National Academy of Science, U.S.A.* that pioglitazone, which was used for treating diabetes mellitus type 2, was useful for resisting the overexpression of special proteins related to proliferation of BC and proven to be effective for killing BC at cellular and animal levels [46]. Furthermore, metformin, which is utilized for treating diabetes, has been also demonstrated by several teams to be helpful for resisting BC [47,48].

Supposing that we only take molecular subtypes into account, the drugs which are essentially useful for preventing malaria and treating hypertension or diabetes would be likely to be ignored by us. Since some patients with BC have pretty favorable reactions to these drugs, we are expected to incorporate these drugs into the aforementioned database and record transcriptomic characteristics or molecular types of patients with BC related to drug reactions, in order that they can be used as references for matching with future patients and selecting treatment plans for them. What is more, all these existing drugs are helpful for us to save billions of U.S. dollars and over 10 years of development cycle as mentioned above.

Nevertheless, the major focus of precision medicine for BC lies in unavailability of enough valid data. With the great support of national governments for scientific medical research, clinicians have had great demands for basic scientific research, which have further aroused medical institutions’ greater concerns about sample collection and preservation. Only if an adequate amount of data are available that it is possible to develop big data, which will be a cornerstone for promoting precision medicine in the future. Furthermore, we have to mention that there is a huge amount of transcriptomic information. To create a transcriptomics-based database about drug reactions of cases, it is necessary to eliminate transcriptomic information which is unrelated to drug reactions in combination with various bioinformatics information [49,50].

**Figure 1:**
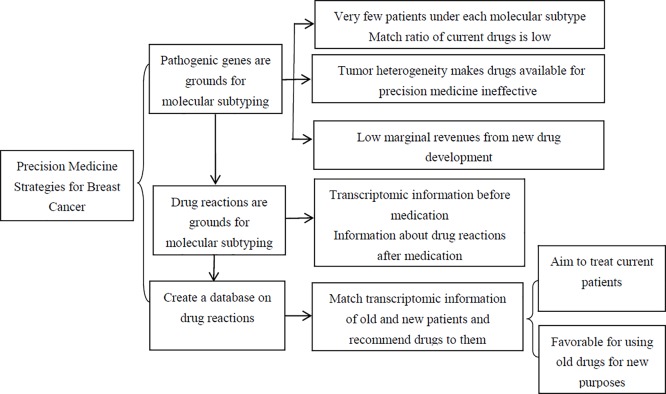
Sketch map of precision medicine strategies for BC

Above all, genomic research has been rather controversial during the development of precision medicine. The precision of proteomics and metabolomics is far from enough. In consideration of deficiencies of precision medicine based on molecular subtypes, we consider that it is effective to make precision medicine possible in the future by performing transcriptomic research to find out changes related to drug reactions and ultimately create a database matching cases on the basis of adverse reactions. In an era of big data, looking for cases to match with all individuals who have demands through data and helping patients identify the best treatment plans will be the preferred individualized treatment strategy (see Figure 1).

## Abbreviations

BC: breast cancer
ER: estrogen receptor
GT198: Genomic Transcript 198
HR+: hormonal receptor-positive
lncRNA: long non-coding RNA
MALAT1: metastasis-associated in lung adenocarcinoma transcript 1
NCI: National Cancer Institute
PDX: patient-derived xenograft
TNBC: triple negative breast cancer
